# Protein homeostasis under heat stress: The role of BiP3, HsfA2, and chromatin remodeling in plants

**DOI:** 10.1093/plphys/kiaf172

**Published:** 2025-05-02

**Authors:** Neeta Lohani, Prateek Jain

**Affiliations:** Plant Physiology, American Society of Plant Biologists; Donald Danforth Plant Science Center, St. Louis, MO 63132, USA; Plant Physiology, American Society of Plant Biologists; Elysia Bio, Raleigh, NC 27606, USA

Global temperature rise is adversely affecting all living organisms, including plants. High temperatures disrupt plant growth, reproduction, and cellular processes, ultimately reducing yield. To develop climate-resilient crop varieties, it is crucial to understand fundamental cellular processes, such as protein homeostasis, which regulates the balance between protein synthesis and degradation (Sun et al. 2021). Protein homeostasis is a “protein quality control” mechanism, ensuring minimal accumulation of unfolded or misfolded proteins.

The primary concern is how protein homeostasis can be safeguarded under elevated temperatures. Under normal conditions, the endoplasmic reticulum (ER) lumen is responsible for a protein quality check; however, excessive heat disrupts protein homeostasis and causes accumulation of misfolded proteins (Liu and Howell 2016). To overcome this, plant cells activate the unfolded protein response that manage misfolded proteins via ER-residing binding immunoglobulin proteins (BiPs), a subfamily of heat shock protein 70 (HSP70) chaperones. Under heat stress, the expression of BiPs is regulated by heat shock transcription factors (TFs) like HSFA2, which further activate several stress sensors and TFs for unfolded protein response (Schramm et al. 2006). Beyond these chaperones and TFs, epigenetic mechanisms like chromatin remodeling can further accelerate or fine-tune the stress response (Cheng et al. 2024). Although these components are partially understood, the mechanism by which BiPs, HSFA2, and chromatin remodelers coordinate protein homeostasis under heat stress remains unclear.

Recently in *Plant Physiology*, Li et al. (2025) demonstrate the intricate network of BiP3, HSFA2, and chromatin remodeling protein CaCHR28 for protein homeostasis during heat stress in pepper and tomato. Building on their earlier work, where they characterized 3 *BiPs*—*CaBiP1*, *CaBiP2*, and *CaBiP3*—in pepper (*Capsicum annuum* L.) and identified *CaBiP3* as having superior activity in abiotic stress-induced ER stress (Wang et al. 2017), the authors now expand their research to examine the role of *CaBiP3* in heat stress. To validate its function, they silenced cabip3 via virus-induced gene silencing (VIGS) and observed increased heat sensitivity in pepper plants (Fig. 1A). Conversely, transient overexpression of CaBiP3 enhances heat tolerance, with plants exhibiting reduced heat damage symptoms and stronger induction of heat-responsive genes.

**Figure 1.**
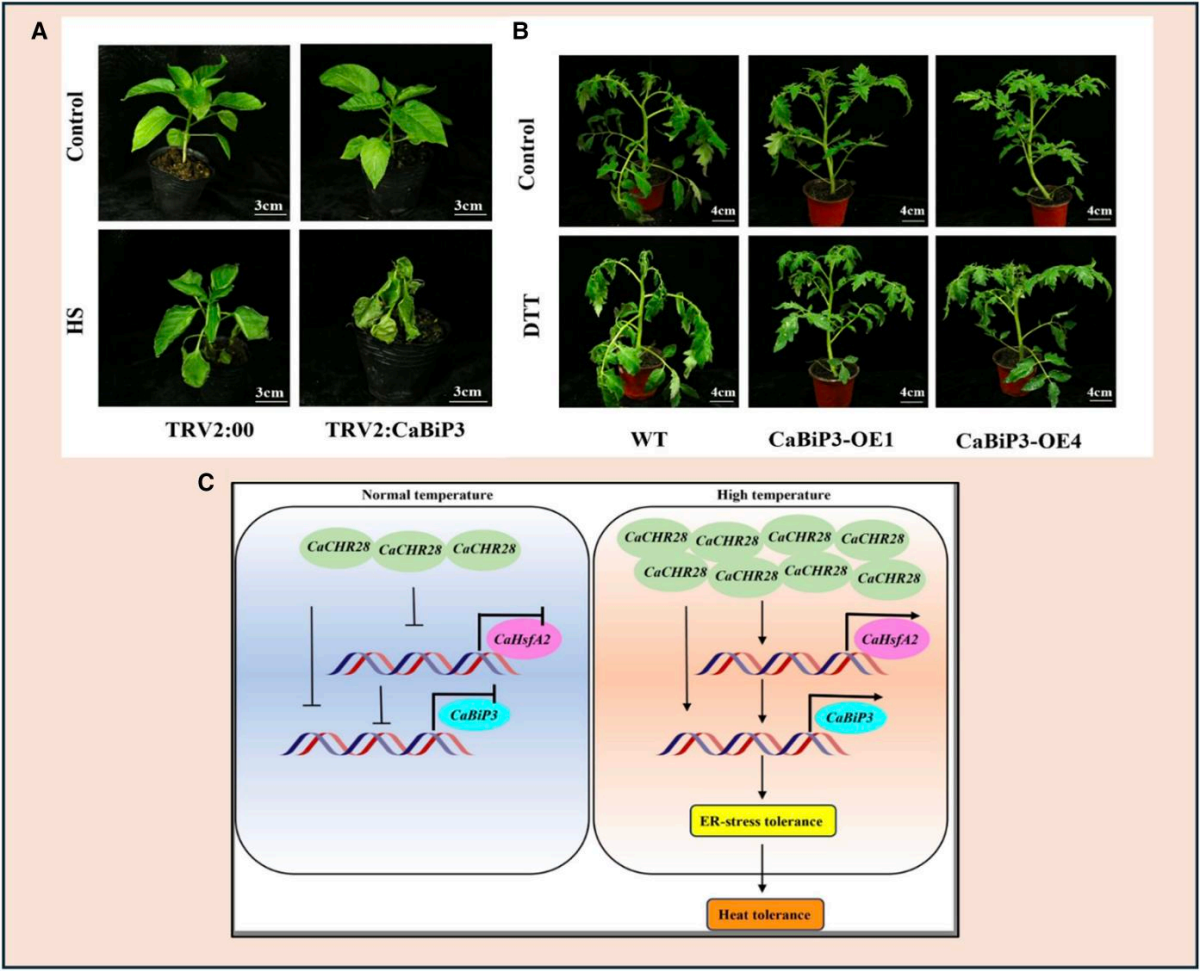
*CaBiP3* positively regulates pepper's tolerance to heat stress. **A)** VIGS silenced *CaBiP3* pepper lines, *TRV2:CaBiP3* have a heat stress phenotype. **B)** The overexpression of *CaBiP3* in tomato plants mitigates the heat stress phenotype induced by dithiothreitol. **C)** A model showing the heat tolerance in pepper mediated by CaCHR28-CaHsfA2-CaBiP3 module.

To investigate the molecular mechanisms and universal role of BiP3, the authors analyzed BiP3 sequences in other plants, including Arabidopsis and tomato. Their analysis revealed a conserved HSP70 domain, essential for protein homeostasis and tolerance under high temperatures. To validate this, they developed CRISPR-edited *slbip3* tomato plants, which displayed heightened heat sensitivity compared to wild type. Overexpression of *SlBiP3* reversed this sensitivity, confirming BiP3 as a key and conserved component of the plant thermotolerance mechanism. Further, transient expression of *CaBiP3* in tobacco leaves showed ER localization, consistent with earlier reports in *Arabidopsis* identifying BiP3 as an ER stress marker (Srivastava et al. 2013). Exposure to an ER stress-inducing chemical (dithiothreitol) resulted in significant damage in *CaBiP3*-silenced pepper plants, whereas overexpression of *CaBiP3* in tomato plants provided higher tolerance (Fig. 1B). Additionally, Li et al. (2025) observed upregulation of ER stress-related genes in heat-stressed transgenic tomato plants, further confirming BiP3’s role in heat stress response via the ER-dependent pathway.

Having established *CaBiP3*’s role in heat stress, the authors next sought to determine its upstream regulators. The authors identified 3 heat shock elements in the *CaBiP3* promoter, and following yeast 1-hybrid screening and DNA binding assays revealed 2 key regulators that converge on the promoter of *CaBiP3*: CaCHR28, and CaHsfA2. They demonstrate that both proteins directly bind to the *CaBiP3* promoter and enhance its expression under elevated temperatures. CaCHR28 employs a dual regulatory strategy—directly binding to the *CaBiP3* promoter while also inducing *CaHsfA2* expression, which further amplifies *CaBiP3* by binding to heat shock elements in its promoter. Under high temperatures, the interaction between CaCHR28 and CaHsfA2 is strongly induced, amplifying the expression of *CaBiP3*.

Further, the authors investigated the subcellular localization of CaCHR28, confirming its role in heat responses, which is vital for plant health under heat stress. Transient transfection in tobacco confirmed CaCHR28 nuclear localization, while functional studies by VIGS showed that silencing *CaCHR28* and *CaHsfA2* severely compromised pepper's heat tolerance. Through methodical genetic rescue experiments, the authors established that overexpressing *CaHsfA2* in *CaCHR28*-silenced plants partially restored heat tolerance, whereas overexpressing *CaBiP3* fully rescued the heat-sensitive phenotypes of both *CaCHR28*- and *CaHsfA2*-silenced plants. This CHR28-HSFA2-BiP3 regulatory module reveals a sophisticated connection between chromatin remodeling, transcriptional regulation, and ER-specific protein quality control, providing deeper insights into how plants coordinate multiple cellular processes to withstand heat stress (Fig. 1C).

The findings by Li et al. (2025) open new avenues for crop improvement in the scenario of climate change. Targeting the CHR28-HSFA2-BiP3 regulatory module could offer an effective approach for developing heat-tolerant crop varieties. Furthermore, the identification of this dual regulatory pathway enhances our understanding of how plants integrate different cellular processes to mount effective responses against environmental stresses. Future research should explore the intricate coordination between epigenetic regulation and transcriptional control in plant stress responses. Further studies should also investigate whether similar regulatory mechanisms exist in other major crop species and assess the potential applications of this knowledge in breeding or engineering heat-tolerant plants.

## Data Availability

No new data were generated or analysed in support of this.
